# Nanopore sensing of protein and peptide conformation for point-of-care applications

**DOI:** 10.1038/s41467-025-58509-8

**Published:** 2025-04-04

**Authors:** Laura Ratinho, Nathan Meyer, Sandra Greive, Benjamin Cressiot, Juan Pelta

**Affiliations:** 1 Université Paris-Saclay, Univ Evry, CY Cergy Paris Université, CNRS, LAMBE, Cergy, France; 2 DreamPore S.A.S., Cergy, France; 3 Université Paris-Saclay, Univ Evry, CY Cergy Paris Université, CNRS, LAMBE, Evry-Courcouronnes, France

**Keywords:** Nanopores, Single-molecule biophysics

## Abstract

The global population’s aging and growth will likely result in an increase in chronic aging-related diseases. Early diagnosis could improve the medical care and quality of life. Many diseases are linked to misfolding or conformational changes in biomarker peptides and proteins, which affect their function and binding properties. Current clinical methods struggle to detect and quantify these changes. Therefore, there is a need for sensitive conformational sensors that can detect low-concentration analytes in biofluids. Nanopore electrical detection has shown potential in sensing subtle protein and peptide conformation changes. This technique can detect single molecules label-free while distinguishing shape or physicochemical property changes. Its proven sensitivity makes nanopore sensing technology promising for ultra-sensitive, personalized point-of-care devices. We focus on the capability of nanopore sensing for detecting and quantifying conformational modifications and enantiomers in biomarker proteins and peptides and discuss this technology as a solution to future societal health challenges.

## Abstract (web summary)

Nanopore electrical detection has shown potential in sensing subtle protein and peptide conformation changes, and its sensitivity makes it promising for point-of-care applications. In this Review, the authors discuss the capability of nanopore sensing for detecting and quantifying conformational modifications and enantiomers in biomarker proteins and peptides, as well as the practicalities and challenges for this approach to be used for clinical and point-of-care diagnosis.

## Introduction

**Fig. 1:**
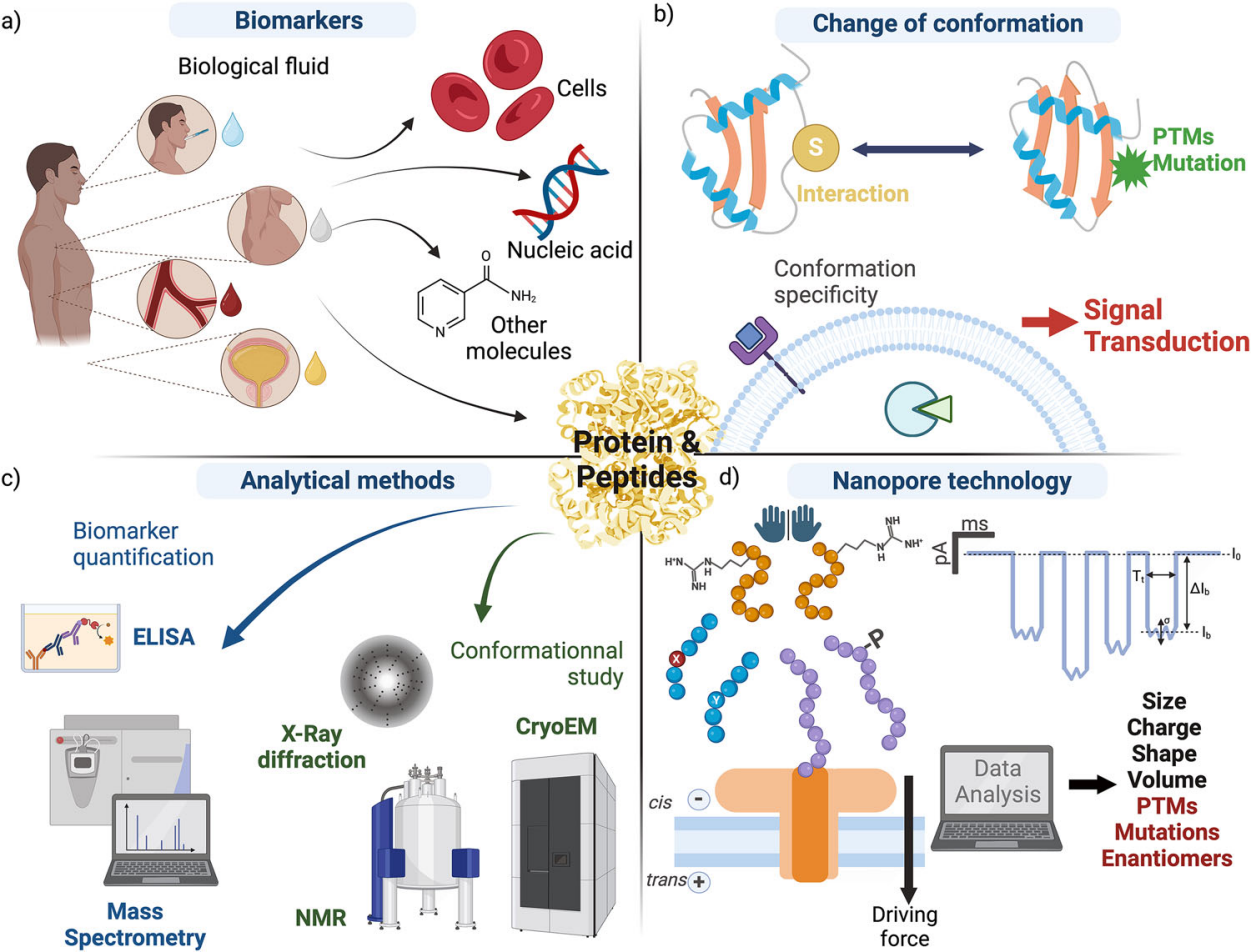
Analysis of peptide and protein biomarker conformations for diagnosis by nanopore. **a** Common biomarkers (cells, nucleic acids, protein and peptides, other small molecules such as vitamins (nicotinamide, vitamin B3) found in different biofluids (examples displayed as drops: saliva (blue), sweat (white), blood/serum (red), urine (yellow)). **b** Schematic representation of a tertiary structure of a protein altered by interaction with a substrate, post-translational modification (PTMs) or mutations, and the role of conformation specificity in receptor or ligand binding. **c** Examples of methods for biomarker quantification (ELISA, Mass spectrometry) and conformational analysis of proteins (X-ray diffraction, NMR, CryoEM). **d** Schematic representation of a nanopore experiment. A single channel (aerolysin) is inserted in a lipid bilayer, and a difference in potential (driving force) is applied by two electrodes in the *cis* and *trans* compartments. Analytes with different amino acid enantiomers, sequence, and/or PTMs driven interact with the pore producing a characteristic drop in the current. Schematic representation of events showing the open pore current (*I*_0_), blockade current (*I*_b_) resulting in a blockade level (Δ*I*_b_) over a dwell time (Tt), and σ (sigma) the standard variation of the event, characteristics of the electrical signal detecting the peptide. Extraction of the characteristic parameters for each signal provides information on analyte size, charge, shape, and volume. Created in BioRender. Ratinho, L. (2025) https://BioRender.com/m45s676.

The current rate of global population growth predicts that there will be ~10 billion inhabitants by 2080, according to the United Nations World Population Prospects 2024. Furthermore, the average age of the population is increasing; by 2080, people over 65 will outnumber children under 18. Since aging brings a variety of chronic diseases, neurodegenerative disorders, or cancer^1^, we need to develop new early diagnostic techniques to improve health management. Early diagnosis can lead to better treatment options, resulting in fewer long-term complications and improved quality of life^1^. One of the tools for diagnosis is biomarker detection and identification from a patient’s biofluid. A biomarker is a defined characteristic that is measured as an indicator of normal biological processes, pathogenic processes or responses to an exposure or intervention^2^. It must not only define a pathology but also be involved in one of the pathological processes^2^. Many factors, such as cell count, nucleic acids, proteins, peptides, vitamins (Fig. 1a), blood pressure, fluid intake, and output monitoring, can be biomarkers.

Although biomarkers have long been used to manage health and disease, recent technological advances have significantly expanded the pool of potentially useful biomarkers, increasing interest in their quantitative detection^3,4^. The early stages of biomarker identification primarily focused on alterations in the sequence of the genetic code. These were sufficient to diagnose and explain diseases arising from congenital and genetic abnormalities, or accumulated DNA damage^5^. However, more subtle effects on physiology and health are mediated by changes to the expression levels of the gene products (proteins and nucleic acids), driven by epigenetic factors, along with other post-transcriptional/translational changes which can alter cellular processes^6,7^. Indeed, many aspects of physiology are regulated not just by protein or peptide amino acid (AA) sequence and expression level^8^, but also by complex assembly, activation, post-translational modifications, as well as global and local conformational changes. For this reason, protein and peptide biomarkers found in biofluids, such as blood, saliva, sweat, cerebrospinal fluids, and urine, are commonly used for diagnosis^9,10^ (Fig. 1a). A diagnostically validated biomarker requires a defined baseline and disease threshold for each patient group (age, sex, ethnicity). Concentrations outside this threshold yield a binary “yes” or “no” results in diagnosis^2^. Efficiency and cost-effectiveness are particularly important in clinical diagnostic assays, with automation and high-throughput methods routinely used^11^. Multiplexing, the detection of multiple biomarkers in a single assay, is useful for parallel measurement of biomarkers routinely screened together^11^. Biomarker levels measured in clinical assays can be influenced by factors such as sample collection and processing protocols, the analytical methods used, and the quality of calibration and standardization applied, reported levels can vary widely. As a result, such assays are subject to industry and national medical agency standardization protocols^2,11^.

Point-of-care (POC) diagnosis is a powerful tool for personalized health management, generating results from minimally processed biofluids within ~20 min. An example is the nasal secretion/saliva COVID-19/influenza rapid antigen tests that detect the high levels of SARS-CoV-2 and/or Influenza viral nucleoproteins produced during infection^12–14^. There are relatively few POC tests currently in use, as their development is hampered by lack of simple, robust, sensitive assays that can be easily used by non-experts. One limitation of POC assays is that standardization of sample processing is difficult to achieve in POC assays. This is particularly problematic for biomarkers that appear in low concentrations or require specialized assays^15,16^.

While current clinical laboratory quantification methods for protein and peptide levels have proven to be valuable tools in managing health, often they do not discriminate between conformational variations in the target biomarker. Increasing information on the relationship between the level and variation in the conformational and/or Post-Translational Modification (PTM) states of target biomarkers and their associated physiology suggests that quantitative analysis of low concentrations of biomarker variants represent a new frontier in diagnostic medicine^17,18^. Since defining and exploiting these new biomarkers requires the development of sensitive, rapid and robust assays, many potential biomarkers have not been identified or fully validated. This is mostly due to the limitations in the detection of specific protein and peptide variants, or their conformations, in biofluids, reducing their use in clinical diagnosis. Many biomarkers are found in very low concentrations (fM) in biofluids, particularly in the early stages of disease, making quantitative detection difficult. These levels often decrease further when considering variants of the target biomarker making detection problematic within the background of additional signals found in complex biofluids. The goal is to develop a POC device that would rapidly and accurately quantify low levels of specific forms of a biomarker, or family of biomarkers, that are subtly altered by sequence, conformation, and/or PTMs, directly in a complex biofluid. In this review, we provide examples of potential biomarkers whose variant forms are linked to different disease states and summarize the current state-of-the-art methods for detecting conformational, PTM and sequence variations in these proteins and peptides. We focus on single molecule nanopore electrical sensing as a powerful tool for quantitative analysis of conformational variety in target biomarkers. In the Conclusion we discuss the practicalities and challenges for this approach to be exploited for clinical and POC diagnosis.

## Background: quantitative analysis of biomarker variants

Protein function is regulated by interaction with other proteins (protein-protein interactions, or PPIs), peptides, nucleic acids and/or small molecules, such that changes in conformation play a pivotal role in biological processes. These are often dysregulated in various pathologies^11,19,20^. Such variations can range from local changes in conformation, electrostatic or chemical properties, around a single or few amino acids, to global conformational changes. Minor alterations such as single AA substitutions, enantiomerization or PTMs can impact local conformation or physicochemical properties that alter the capacity to bind to receptors^21,22^, ligands, or ions, as well as the ability to oligomerize^23^ or undergo self-assembly and aggregation^24^, potentially leading to biological malfunction^19,20^(Fig. 1b). These stable or transient interactions play a role in enzymatic activity, metabolism regulation, and signaling pathways important for physiological regulation and disease pathology. Consequently, differentiation of variation in local protein/peptide conformation, single AA changes or PTM state could be extremely useful in biomarkers analysis.

Numerous examples of pathologies are linked to dysregulation introduced by conformational changes in proteins and peptides^25^, including those important for homeostasis mechanisms such as the self-assembly processes in clotting^26,27^, or caused by self-misfolded protein/peptides aggregation-based pathologies such as Alzheimer’s, Parkinson’s, or Crusztfeld Jabob diseases^28–30^. For instance, mutations in p53 protein, which is involved in cancer, can lead to structural changes that alter function^31^. While there are many methods to define conformational variation, few of these are sufficiently quantitative for clinical use. Detecting the subtle changes that arise from a single AA modification or change in PTM state in a biofluid is challenging^32^, since the signal for the target molecule is difficult to differentiate from background signals generated by other component molecules. Indeed, given the difficulty in sensing small local changes within a complex environment, the role that such small changes have in regulating homeostasis is not well understood. We next summarize the current state-of-the-art technology in defining conformational variety in proteins and peptides and discuss their current utility for biomarker detection.

### Current methods for biomarker quantification and conformational analysis

Mass spectrometry (MS) (Fig. 1c) is used to identify potential biomarkers between matched patient and control groups followed by statistical analysis of the results to define a biomarker profile that is specific to a single pathology^33^. The targeted biomarker can be quantified by employing an unlabeled^34^ or isotope labeling approach^35^, however, this technique needs at least 10^6^ copies of the same protein for detection^36,37^. To enhance sensitivity and specificity, samples are typically pre-processed by chromatography to make detection of small changes in concentration easier^33^. While this technology is sensitive enough to detect changes in AA and PTMs that affect the mass-to-charge ratio of a protein or peptide, the results are averaged from bulk analysis, potentially missing rare events that could better explain disease pathology. Additionally, MS analysis is unable to differentiate between variants that have the same m/z ratio, such as phosphorylation or sulfation PTMs of tyrosine, different conformations or enantiomerization^38^. This approach also requires expensive equipment, highly trained operators, and a very high analyte concentration, limiting the utility and cost-effectiveness in routine clinical diagnostics.

Immunoassay technologies, such as Enzyme-Linked Immunosorbent Assays (ELISA), or fluorescent immunoassays are an affinity binding-based approach, commonly used for clinical biomarker quantification^9,33^ (Fig. 1c). These assays use antibodies (Abs) to specifically detect targeted proteins or peptides, even at low concentrations (fM)^33^. Fluorescent assays can be multiplexed by using magnetic beads and/or fluorophores detected at different wavelengths^11^. Recently developed Olink or proximity extension assays combine antibody specificity with PCR sensitivity, using Ab-oligonucleotide conjugates, allowing detection via real-time PCR. This technique is highly sensitive (pg/ml or 10^4^–10^5^ molecules), compatible with biofluids, and easily multiplexed with DNA barcode sequences^11,39^. When implemented as POC tests, membrane-bound, lateral-flow immunoassays (for instance for Influenza virus) usually require high protein concentration (~10^9^ molecules/mL, 10^8^ molecules assuming 100 μl test volume, similar to ELISA)^14,40–42^ meaning infections with low viral load are not detected.

Antibodies can be created and engineered to bind to specific epitopes in the protein or peptide of interest^43^. Two main types of epitopes exist: the continuous epitope, in which a small part of the primary AA sequence (8–12 AA)^44^ is bound by the antibody; and the discontinuous/conformational epitope, where the AAs may be far apart in the primary sequence but are in spatial proximity in the mature protein^45^. Consequently, immunoassays are limited by the inherent properties of antibody–epitope interactions. For example, Abs may be unable to detect changes in the primary sequence or minor changes in conformations if they are not present on the epitope, leading to cross-reactivity and the detection of different variants in addition to the target biomarker. This is particularly problematic for assays detecting cleaved peptides (the target biomarker). The inability to discriminate these products from the source protein they derive from skews the final measurement^46,47^. Alternatively, changes in the epitope can reduce the binding efficiency of the Ab leading to false negative assays. For instance, even small changes in the SARS-CoV-2 nucleoprotein can prevent the antibodies used in the POC test from binding, creating false negative results^48^.

Traditional methods for defining conformation are crucial in understanding how protein conformation relates to function, including techniques such as NMR, X-ray crystallography, and CryoEM (Fig. 1c). Each of these techniques has different degrees of resolution as well as limitations. For example, they need an abundance of proteins, high sample purity and/or cannot define conformational dynamics^49–51^.

Vibrational spectrometry techniques, such as Raman and infrared spectrometry, are advantageous for biomarker detection as they detect conformational changes in biomolecules^52–54^. These non-invasive methods enable quick and accurate biomolecule detection, making them promising for diagnosing diseases like cancer, while reducing patient risks by eliminating the need for markers or dyes. However, there are several drawbacks to their use in the clinical setting. The instruments are highly sensitive, expensive, and generally non-portable, requiring expert operators and extensive calibration for quantitative measurements^55,56^. The signals generated can be weak, limiting use to biomarkers that are present at high concentrations in biofluids^57^. Additionally, biological tissue fluorescence can interfere with measurements and the limited tissue penetration of the lasers restrict use to accessible areas^58^.

There is a growing interest in single-molecule techniques to study the conformational heterogeneity of proteins or peptides in solution. These methods are ~5 orders of magnitude more sensitive than the bulk methods described above, however, since statistical power is related to sample size, 10^2^–10^3^ molecules are required for robust analysis^59,60^. Examples include Single-molecule Förster Resonance Energy Transfer (smFRET)^61,62^ or Atomic Force Microscopy – Single-molecule force spectroscopy (AFM-SMFS)^63–65^ (Table 1). While these permit the definition of conformation at the single molecule level, they require a highly purified or pre-treated sample precluding their use on biofluids. The equipment is expensive, requiring highly trained staff and reducing feasibility for use in clinical diagnosis.

**Table 1:** Analyzed features, Spatial resolution, labeling requirement and limitations of single-molecule techniques: single-molecule Förster Resonance Energy Transfer (smFRET)^62^, Atomic Force Microscopy—Single-molecule force spectroscopy (AFM-SMFS)^63–65,219^ and Fluorescence correlation spectroscopy (FCS)^66,67^

Single molecule technique	Analyzed features	Spatial resolution	Labeling requirement	Limitations
smFRET^62^	- Kinetic determination - Molecular dynamics (ns to s timescale) - Conformational analysis	10–100 Å distance in vivo and in vitro	Fluorophore labeling	- Can’t define local conformation changes - Difficulty in site-specific labeling - Not suited for quantification
AFM-SMFS^63–65,219^	- Indirect structural information and conformational changes induced by protein/ligand binding	5–10 Å	Linker to molecule of interest	- Highly purified sample - Not suited for quantification
FCS^66,67^	- Molecular dynamics - Diffusion coefficient - PPI- Analyte quantification	50 Å in vivo 10 Å in vitro	Fluorophore labeling	- No detection of PTM and AA mutations if it does not alter protein dynamics

Fluorescence Correlation Spectroscopy (FCS)^66,67^ has the potential for use in single-molecule biomarker detection, combining quantification and conformational analysis since it has been used to accurately quantify biomarkers^68–70^. However, to quantify analytes directly from a biofluid, this technique needs labeled antibodies targeting the biomarker of interest, with limitations similar to ELISA techniques^70^.

### Nanopore sensing technology

As a single-molecule, real-time electrical detection method that operates in the nanoscale range (nm or 10 Å scale), nanopore sensing is a powerful technique in the analysis of individual molecules. First described in 1986, nanopore technology was used as a molecular counter, showing the proof of concept that polymers could interact with protein nanopores creating quantifiable characteristic current blockades^71^. In 1996, Kazianowicz et al. showed the electrophoretic transport of DNA through nanopores^72^. This technique was developed for ultra-fast DNA sequencing by Oxford Nanopore Technologies and is now used in the clinical diagnosis of unknown viral infections and has been deployed by clinicians/epidemiologists in the field^73^.

Nanopore sensing was later extended to study other types of biomolecules. Several pioneering studies permitted the understanding of protein and peptide interaction with the nanopore: capture and transport dynamics^74,75^ allowed the detection of proteins and peptides; conformational^76^ and PPI^77^ analysis demonstrated that nanopores can detect changes in conformation and methods to control polypeptide chain unfolding and translocation speed^78–80^ were defined. Recently, these advances have been applied to protein sequencing, as well as peptide and protein biomarker detection^37,81–85^. Given the proven success of nanopore nucleic acid sequencing, this approach could be a key tool in the development of clinical/POC diagnosis technology.

Nanopore experiments use an electrical force to drive molecules through a single nanometric scale channel. Two chambers, filled with an ionic solution, are separated by a lipidic membrane containing a single protein nanopore. Upon applying a potential difference, ions flow through the pore towards the oppositely charged electrode, creating a characteristic open pore current (*I*_0_) (Fig. 1d). For a known applied voltage, *I*_0_ is proportional to the buffer conductivity (*κ*), and the channel dimensions of area (*A*) and length (*l*_pore_): *I*_0_ ∝ (*A*/*I*_pore_) × *κ*.^86,87^ This means that the *I*_0_, and thus the signal to noise ratio (S/N), scales inversely with pore length^88^. Interaction of an analyte molecule with—or translocation through—the nanopore will partially block the ions going through the pore, resulting in a drop in the measured current defined by the blockade level (*I*_b_) or blockade amplitude (Δ*I*_b_). Each blockade event is also classified by the dwell time/blockade duration (*T*_t_) and the noise (standard deviation, *σ*) of the *I*_b_^89^ (Fig. 1d). Two types of events are normally observed for every analyte. Short, or bumping events (dwell times <100 μs) that result from transient interaction of the analyte with the pore^90^, are too short for accurate analysis of the event parameters and not useful in analyte discrimination. Conversely, longer events (>100 μs) where the analyte engages the pore opening, create blockade events which are unique to the individual molecule interacting with the pore. Indeed, each molecule of the specific analyte produces highly reproducible events, defined by the blockade parameters, allowing classification into discrete populations based on their uniquely identifying combination of blockade parameters^91–93^. The reproducibility of these event parameters is directly related to the physical properties of a particular analyte that define its interaction with the sensor channel^94,95^. Indeed, blockade levels are proportional to the relative volume of the pore occupied by the analyte and transient binding interactions between the pore and the analyte^96–98^. The dwell time is influenced by the charge of the analyte, the driving forces and any interactions with the pore. Finally, the frequency of the interaction of a particular analyte with the pore is related to the concentration of the analyte in solution^75,99^.

Nanopore sensing of protein and peptides introduces additional challenges compared with the sequencing of uniformly highly negatively charged nucleic acid chains, which are driven through the pore by electrophoretic forces. Protein and peptide chains can contain up to 20 different core amino acid monomer units, leading to a large diversity in size, shape, hydrophobicity, net charge, and conformational flexibility. Nanopore approaches must be tuned to maximize sensitivity to the properties of the target protein/peptide analyte, with size and net charge being the most important consideration for designing sensing systems.

Molecules are driven toward the pore by two main forces: the electrophoretic force (EP) and the electro-osmotic flux (EOF). Assuming the electrolyte solution, the membrane thickness and the applied voltage remain the same, the EP is dependent on the overall charge of the analyte, while the EOF is predominantly determined by the internal charges of the nanopore tunnel^100^. As with all protein surfaces, the charged AA residues on the wall of the internal channel in a protein nanopore interact with counterions (ions of the opposite charge) in the electrolyte solution. These form a thin layer, along with their associated water molecules (hydration shell), that facilitate protein solubility. While these interactions become more complex within the confined environment of the channel, application of an electric field facilitates the displacement of these surface counterions, and their hydration shells, towards the oppositely charged electrode. The net charge of a nanopore depends on the pH of the electrolyte and the overall sum of positive and negative charges from the AAs lining the tunnel. The net EOF refers to the total movement of positive and negative counterions under the applied electric field. When the distribution of the AA charges on the pore surface contribute to a non-neutral net charge, the pore preferentially interacts with counterions that are oppositely charged to the AAs that predominate on the surface of the channel. These ions then permeate more easily through the pore^89^ towards the oppositely charged electrode. Hence, if there is a net accumulation of counter ions inside the nanopore, a directional flow of water and ions is generated, called electroosmosis flow. The shape and size of the nanopore along with the type and ratio of the cations and anions going through the pore defines the orientation and intensity of the EOF^87,89,100^. Modulation of these driving forces can augment the efficiency of capturing analytes at low concentrations as well as improving the resolution of analyte-pore interaction signals. Understanding these forces is currently a significant challenge for designing nanopore systems with high sensing efficiency for low concentration analytes, high-resolution, feature-rich analyte signals, that can discriminate the target analyte from the background signals of complex biofluids.

For charged analytes, the overall driving force is dependent on both the EOF and the EP, which, based on the ion selectivity of the pore and the analyte charge, are synergistic or antagonistic. If the analyte has the same net charge as the pore-selected ion, the EP and EOF would be synergistic. These combined forces could promote the detection of such analytes at very low concentrations by increasing event frequency. Another method of modulating this driving force is to alter the charge on the analyte. This can be achieved by attaching the analyte to a molecule that is highly charged at pH 7.5 (eg. poly-arginine chain^101^ or DNA/RNA^102^) increasing the EP to drive the protein or peptide towards the pore.

Alternatively, for neutral or weakly charged analytes, the main driving force for nanopore interaction is EOF. The EOF in protein pores can be modulated by engineering the amino acid residues on the inner surface of the nanopore or by altering the type of ions in the electrolyte solution. Indeed, modulating the EOF can increase the sensitivity and selectivity of the pore by adjusting transport and translocation speed as well as capture efficiency. Mutations in biological pores altering EOF change the interaction dynamics of analytes, leading to improved detection^103–105^. These strategies have been utilized in several studies as described below.

Outside the driving force, pore sensitivity can be improved by increasing the confinement of the analyte inside the pore. One method of enhancing confinement is to slow the interaction of the analyte with the pore (T_t_). For example, when the analyte has the opposite charge to the pore-selected ions, the EOF and EP would be antagonistic. Tuning the balance of these opposing forces can increase the resolution of analyte signals by slowing the translocation time, thus increasing the T_t_ and/or increasing the resolution of the blockade level resulting, in a more defined analyte blockade signal^104^. This can also be mediated by transient interactions between the chemical groups lining the pore and those on the surface of the analyte. These interactions can be modulated by changing the apparent diameter of the pore, through mutations, chemical modification, or molecular adaptor^27,106–110^. Confinement, where the analyte blocks ~75% of the pore diameter^111^, facilitates transient interactions and/or creates friction, prolonging the pore interaction time T_t_ and increasing the S/N for improved sensitivity. Such confined interactions are highly sensitive to local changes to the conformational and surface physicochemical properties of the analyte. This underlies the resolution of nanopore sensing for very small changes, such as single AA mutations, PTMs, conformational changes, or AA enantiomerization (Fig. 1d).

The high sensitivity and broad analyte sensing capability of this technique has allowed researchers to not only sequence DNA and RNA depending on the blockade of the bases as they translocate through the pore^112^ but to also study the structure^25,32,76,77,113–122^, sequence^32,91–93,101,119,123–126^, or PTM^25,27,95,127–132^ states of proteins and peptides interacting with the pore. Since many short peptides are conformationally flexible, this technique also has the resolution to discriminate between peptides with different conformations: those with one or more defined structures and conformationally dynamic unfolded peptides^78,133,134^. This was particularly well demonstrated by the identification and discrimination of β-hairpin^135^ and α-helical^136^ peptides.

Different methods have been used to characterize analytes by nanopore sensing: threading, slow translocation of a long molecule such as for DNA sequencing^137,138^; fingerprinting, which identifies a specific event characterized by the confinement of an individual analyte molecule (volume, conformation)^139^; and molecular trapping, which involves capturing the molecule inside the pore for a long time in order to observe the real-time fluctuations in current resulting from intrinsic molecular dynamics or the interaction of the analyte with ligands or ions^107,140^.

Nanopore sensors can be designed to detect and quantify heterogeneous biomarkers. For this purpose, the pore size is crucial for optimal detection, and the diameter of the channel should be no more than 1.5 times larger than the hydrodynamic radius of the analyte^111^. This ensures efficient confinement of the analyte inside the pore for high-resolution signals permitting the discrimination of differences in conformations or physicochemical properties^111^. Larger analytes (>5 nm diameter) are usually detected by solid-state nanopores that can be reliably fabricated to ~5 nm^141^. These are made of various materials, including SiN, polymers, quartz, or graphene, which allow the detection and study of large macromolecules such as protein aggregates, protein complexes, and DNA-protein interactions^141^. While fabrication techniques allow the diameter of the pore to be tuned for different-sized analytes, solid-state pores are difficult to fabricate reproducibly for small diameters. In comparison, biological nanopores allow for directed tunability of the internal properties in the sub-nanometer scale, reducing the resolution for discrimination of subtle changes in protein and peptide properties. The natural variety of different protein nanopores provides a library of nanopores with constriction diameters ranging from 0.7 nm for OmpF, 1.5 nm for Aerolysin, 5.5 nm for PlyAB^142^ and up to 12 nm for Poly(C9)^143^. Such protein pores are more suitable for smaller molecules and have been used to characterize many different analytes depending on their globular size (<5 nm diameter) and occupied volume inside the pore.

### Direct or indirect detection by nanopore: improving sensitivity to size and conformation

**Fig. 2:**
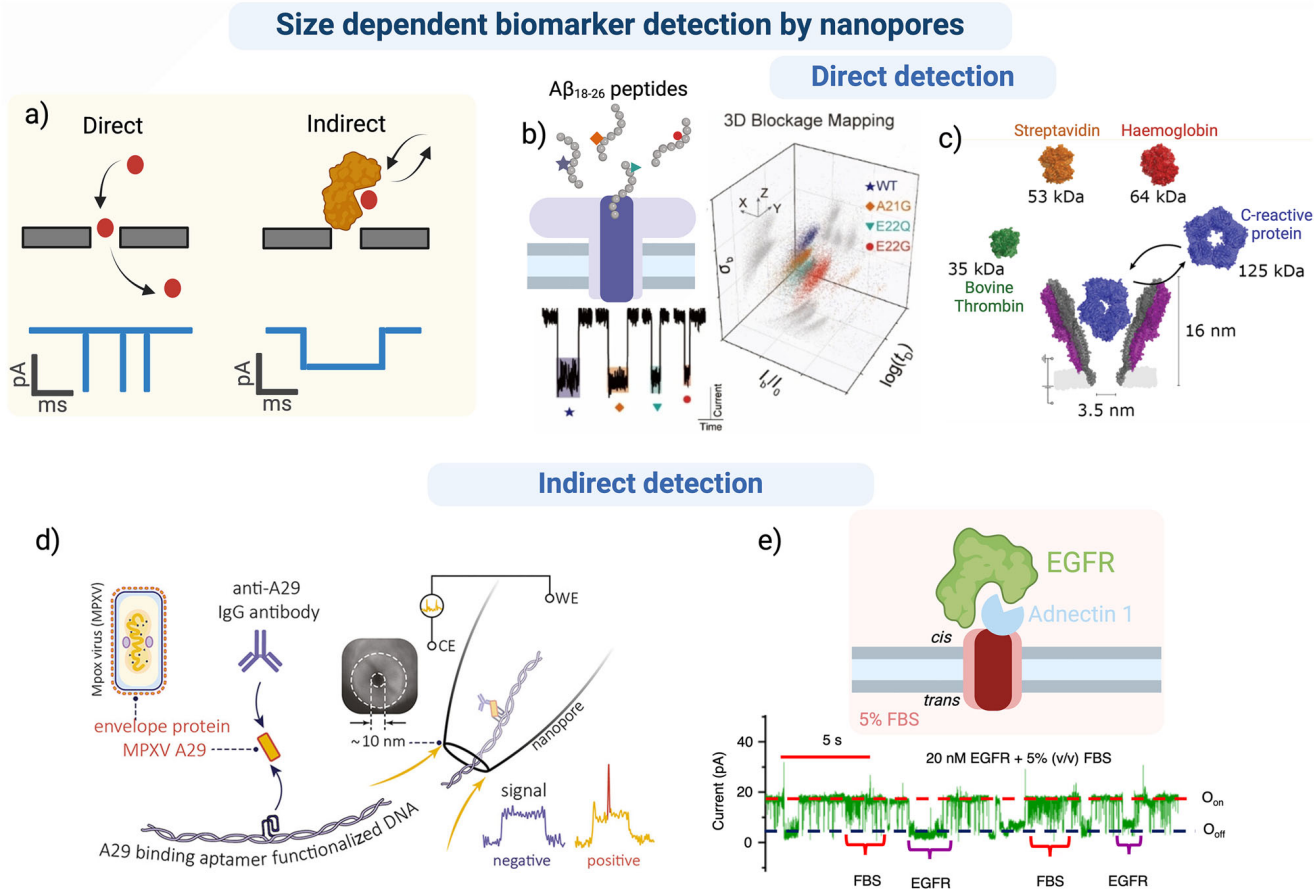
Size-dependent biomarker detection by nanopores. **a** Schematic representations of current blockades that related to relative ratio of analyte size to pore diameter in direct and indirect detection methods. **b** Direct discrimination of Aβ peptides that differ by one amino acid with unique blockade characteristics using an Aerolysin nanopore in 1 M KCl, 10 mM Tris, 1 mM EDTA, pH 8. Figure adapted from Angew. chem., Xin et al. © 2022 Wiley_VCH GmbH^149^ **c** Direct detection and characterization of a several proteins with different shapes and sizes: Bovine Thrombin (BT: 35 kDa), Streptavidin (SA: 53 kDa), Hemoglobin (HG: 64 kDa) and C-reactive protein (CPR: 125 kDa) with a YaxAB nanopore in 150 mM NaCl, 150 mM Tris, pH 7.5. Figure adapted from ACS nano, Straathof et al.^150^. **d** Principle of the detection of the viral envelope protein MPVX A29 bound to an antibody and functionalized aptamer with a nanopipette. Figure adapted from Nano letters, Cai et al.^102^. **e** Schematic representation of a tFhuA nanopore engineered to attach an Adnectin 1 monobody binding EGFR in 5% FBS. Current traces and histogram of the blockade level of the EGFR-pore interaction in 5% FBS. Figure adapted from Nature Com., Ahmad et al.^146^. Created in BioRender. Ratinho, L. (2025) https://BioRender.com/s75n220^147^.

Single-molecule electrical detection with nanopores can distinguish individual analytes either directly or indirectly. If the pore diameter is significantly larger than that of the analyte, the molecule may translocate quickly through the pore, resulting in a short blockade event (Fig. 2a). Event information is increasingly lost as the blockade amplitude decreases and the speed of the analyte translocation approaches the time resolution of the detection electronics. Additionally, in a complex environment, the high quantity of different molecules can make it challenging to specifically detect individual analytes without significant tuning of the sensor. Consequently, some researchers have opted to use indirect techniques. These rely on specifically inducing a volume increase in the target analyte, creating deeper, more defined blockade levels, and/or on increasing the charged analyte to modulate the driving force. For instance, an affinity approach, using a specific molecular binding partner can bind to a specific analyte in a mixture and increase its size to improve detection. Often, such binding also increases the overall charge, modulating the EP driving force, increasing the capture efficiency, specificity, and sensitivity. This can also enable the bound target molecule to be confined in the pore for longer periods of time, useful for quantitative detection of target analytes at low concentrations, increasing sensitivity and generating feature-rich blockades (Fig. 2a).

In an example of indirect detection, Cai et al. used a quartz nanopipette (10 nm in diameter) to detect and quantify the MPXV A29 protein, an envelope protein present on the surface of the Mpox virus, when bound specifically to both an antibody and a DNA carrier^102^. At physiological pH, the highly negatively charged DNA introduced a strong EP driving force that efficiently translocated the analyte/Ab complex^144^. This resulted in a specific event with different blockade parameters than those for the carrier alone (Fig. 2d). This method was used to quantify the protein (10^3^ molecules) directly in a biofluid (human serum or saliva spiked with 1 nM target protein, followed by dilution to 5% in electrolyte buffer). Since the detection of A29 protein only occurs in the presence of virus, this work shows the potential utility for clinical diagnosis. Indeed, several rapid antigen tests for Mpox A29^145^, with similar sensitivity to POC tests for influenza, are commercially available, although, none have, as yet, been approved for clinical use.

Movileanu’s group used a tFhuA protein nanopore (2.6 nm in diameter), engineered to be conjugated to different nanobodies capable of specifically detecting different biomarkers/biologically relevant proteins^146^. For example, EGFR was detected in a biofluid (5% fetal bovine serum spiked with 20 nM EGFR) when bound to pore-immobilized monobody Adnectin 1 (Fig. 2e). Zhang et al. used protein engineering and bioorthogonal modification of a ClyA nanopore (3.3 nm constriction) conjugated to multiple Ty1 nanobodies capable of detecting the Spike protein from SARS-CoV-2 in a model biofluid (BSA, 6 μM with 2.3 nM spike protein)^147^. This further shows that this technology could be used as a diagnostic tool for detection and quantification of low concentrations of biomarkers directly in biofluids.

While nanopore-based detection of affinity-bound target analytes is highly sensitive, they suffer from the same limitations as immunoassays, described above. Consequently, direct detection is preferable. Use of pores similar in size to the target analyte increases confinement within the pore without the addition of larger binding partners (Fig. 2a). For small analytes, such as peptides of less than 50 amino acids, narrow diameter pores such as Aerolysin (AeL), are required. With a diameter of 1.6 nm, AeL is particularly useful for the direct detection of small peptides (5-20 AA), as well as for sensing the translocation of long unfolded polypetide chains of 370^75,148^, 539, and 1078 residues^99^. For example, Xin et al. used an AeL nanopore to detect and discriminate between Aβ peptides carrying different single AA mutations^149^ (Fig. 2b). As described above, multiple pores with different internal dimensions are often used to characterize different-size analytes. However, the Maglia group exploited the YaxAB pore^150^, to capture different-sized proteins, from the 35 kDa bovine thrombin (∼4.5 nm diameter), to 125kDA for the C-reactive protein (∼9.5 nm diameter). This cone-shaped pore allowed for confinement of the different-sized proteins at different levels inside the pore, creating distinct blockade levels that discriminated between the proteins with high resolution^150^(Fig. 2c).

## Nanopore technology for quantitative conformational sensing

Nanopore technology has shown the sensitivity in detection and capability for quantitation of target analytes at low concentrations (pM), with as few as 10^3^ molecules^60^, even in complex environments such as biofluids^102,114,115,119,146,147,151–154^. Here, we review the application of nanopore technology, particularly the use of confinement, to detect differences in the conformation of proteins and peptides that are potential biomarkers. We focus on examples provided by the most recent work using protein nanopores that highlight two main areas of detection: real-time measurement of conformational dynamics and the discrimination between slight changes in local conformational and/or physicochemical properties that arise from single amino acid substitution, addition of PTMs and/or inclusion of different enantiomers of the same amino acid.

### Real-time kinetics by conformational sensor

**Fig. 3:**
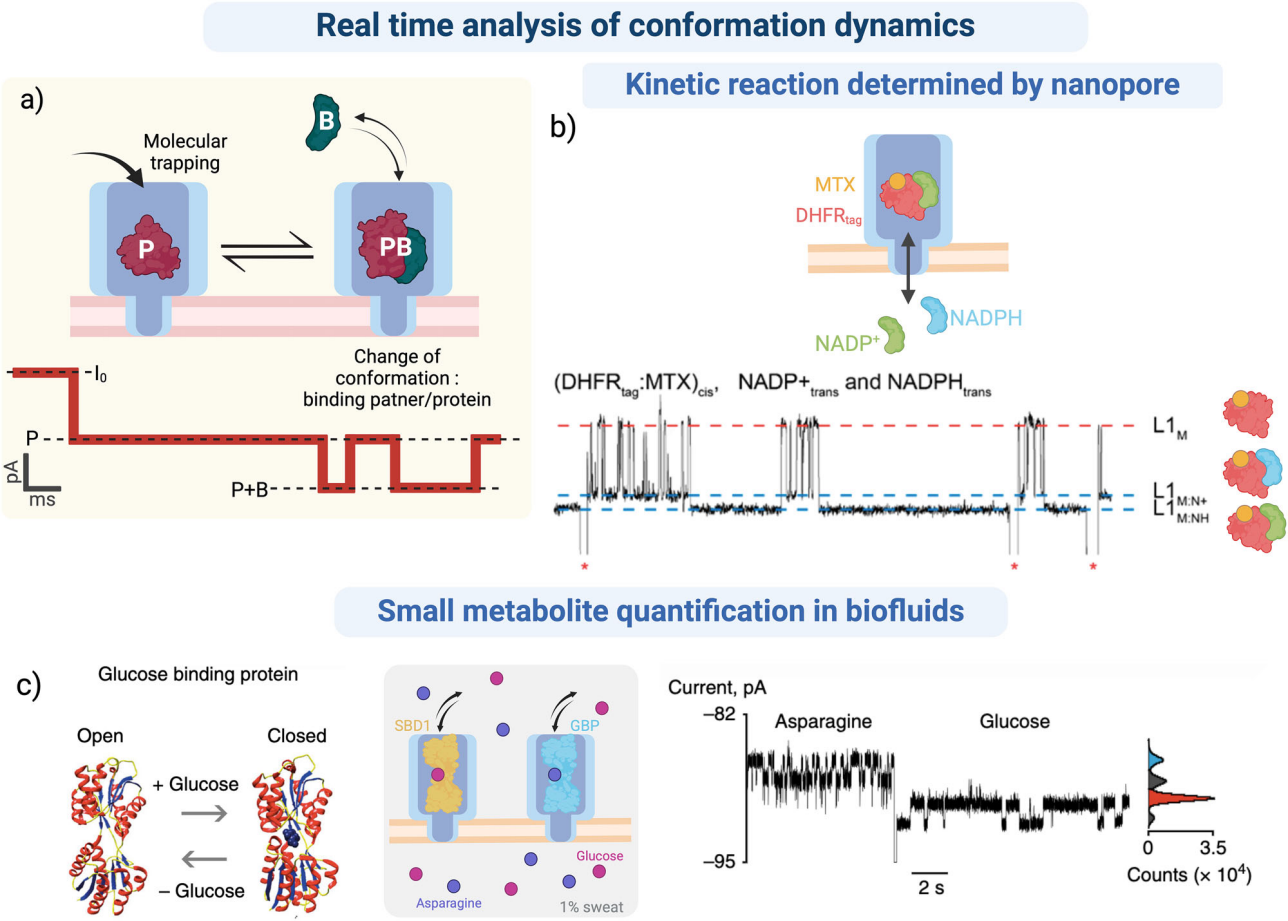
Real-time kinetic analysis with a conformational sensor. **a** A protein (P) is trapped inside a pore by molecular trapping. Partner binding (B), results in a complex with a different conformation. The change in pore volume occupied by the complex or a change in conformation can be detected by a shift of blockade level (P + B) in real time. **b** ^160^Current trace, in real-time, of the capture of an engineered *E. coli* dihydrofolate reductase (DHFR_tag_) complexed with methotrexate (MTX), free or bound to NADPH or NADP+ inside a ClyA nanopore in 150 mM NaCl, 15 mM Tris HCl, pH 7,5. Reprinted (adapted) with permission from Soskine, M., et al. Single-Molecule Analyte Recognition with ClyA Nanopores Equipped with Internal Protein Adaptors. *J. Am. Chem. Soc*. **137**, 5793–5797 (2015). Copyright 2015, American Chemical Society^113^. **c)** Current trace of the capture of SBD1 (substrate binding domain 1) and GBP (glucose binding protein) in a ClyA nanopore unbound, and bound to respectively, Asparagine and Glucose in a 100-fold dilution of sweat in 150 mM NaCl, 15 mM Tris HCl, pH 7,5. Figure adapted from Nature Com. Galenkamp et al.^114^. Created in BioRender. Ratinho, L. (2025) https://BioRender.com/v49u400.

Many disease pathologies may be linked to altered enzymatic reaction kinetics, PPIs, or ligand binding that arise from changes to the AA sequence or PTMs^155–157^. Nanopore technology that traps an enzyme or protein within the pore allows for real-time analysis of changes in conformation. Upon addition of ligands, the binding event can be monitored by the variation in blockade level caused by the subsequent change of volume or conformation of the complex (Fig. 3a). This permits the determination of the kinetic interaction parameters for enzyme-substrate binding or for other protein binding events (complex formation, oligomerizationetc), where such changes could be a biomarker for pathologies like cancer^158–160^ or Alzheimer’s^161^. There are very few techniques that can directly study, in real-time, single-molecule enzyme activity or binding events in biofluids. Most common methods use bulk measurements of changes in substrate or product quantities^162^, or complex composition^159^. Real-time single-molecule conformational analysis, in nanopore confinement experiments, could help define the different conformational states available to each protein, the relative occupancy of these states and the role they play in regulating cell processes. This would allow a deeper understanding of the impacts of local or global changes in conformation due to mutations or PTMs that are linked to different disease pathologies.

One example is the ubiquitination reaction which plays a key role in regulating protein degradation and has been investigated with nanopores by several groups^163–165^. Using a ClyA nanopore, Maglia and coworkers measured the ubiquitination reaction of the ubiquitin-carrier E2 enzyme^163^. They were able to discriminate between non-ubiquitinated E2 and conjugation of one or two ubiquitin molecules to the enzyme. This could be the key to following, in real-time, ubiquitination levels of a target protein.

The same group studied the binding of NADPH, NADP+ to the complex of *E. coli* Dihydrofolate Reductase (DHFR) bound to Metothrexate (MTX). DHFR is often used as a model protein adaptor to study enzymatic reactions^121^. Confinement of DHFR-MTX within the ClyA nanopore was facilitated by increasing the EP driving force (at negative applied voltages) by addition of a biologically inert polylysine tag. The authors detected real-time changes in conformation upon the binding of NADPH or NADP+ (Fig. 3b), discriminated between the binding of the two ligands and defined the kinetic constants of the interaction^113^. The same group has mapped the conformational variants and kinetic interaction parameters that exist for free DHFR, DHFR-MTX, and each of the ligand-bound complexes^120^. This tool could help study altered enzymatic reactions and link them to pathologies. Other nanopore studies indirectly track enzyme activity by measuring the molecular product or modified proteins, to determine enzymatic constants that could be considered biomarkers^164,166–168^.

Nanopore experiments could also help define real-time PPIs crucial in regulating many biological processes. Thakur and Movileanu used a tFhuA nanopore engineered to attach a specific protein receptor to monitor the real-time transient interactions of the target protein with the nanopore-receptor complex^77^. This type of approach could be implemented in diagnosis to highlight the difference in binding affinity between protein binding partners involved in disease pathology. Nanopore sensing of conformation change in a protein was exploited by the Maglia group to quantify small analytes in biofluids^114,115^. Changes to the conformation of Substrate Binding Domain 1 or Glucose Binding Protein upon binding to either glucose or asparagine, respectively, were detected by changes in the blockade levels (Fig. 3c). The analysis of the number of current variations permitted the quantification of these small analytes in 1% sweat^114^. This technology could allow accurate asparagine measurement as a potential biomarker for brain damage, stroke, or Parkinson’s disease^169^. Using the same principle, Kwak et al. measured the activity of neuraminidase (NA), a glycoside hydrolase found on the surface of Influenza A viruses. They achieved this by measuring the increase in galactose concentration and monitoring changes to this activity in the presence of various drugs. This research shows that electrical detection by nanopore could be valuable in POC technology for monitoring NA activity and rapid definition of drug resistance for selection of personalized treatment^122^.

The Long group studied the binding pathways of variants of the spike protein from SARS-CoV-2 to the ACE2 enzyme. This interaction permits cellular invasion and could be an indicator of infectivity^170^. They anchored homologous spike proteins from different viral strains to a nanopipette, so that the binding of soluble ACE2 increased the overall volume of the complex, increasing the blockade level. This data determined the binding affinity of each variant to ACE2, with Omicron S having the highest affinity consistent with its comparative increased infectivity^116^. This approach could be used to define the binding affinity of spike protein from new viral variants to ACE2 as well as testing the efficacy of vaccine antibodies for neutralization of these variants.

The use of protein nanopores involves very fragile and short-lived lipid membranes, and while solid-state nanopores are more durable, they are less reproducible. The Dekker group engineered a solid-state nanopore that permits the trapping of macromolecules for an extended period. The NEOtrap consists of a SiN nanopore on which a DNA origami molecule is docked. This created a strong EOF due to the permeability of the origami structure and the highly negatively charged DNA at neutral pH. The increased force helped capture the target proteins within the nanopore cavity blocked by the docked origami DNA. Proteins like Clpx, HSP90, or Avidin were confined inside the pore for hours during which changes in electrical signals defined, in real-time, the conformational landscape explored by the targeted proteins^117^.

### Sensitivity of single amino acid mutation-induced changes

**Fig. 4:**
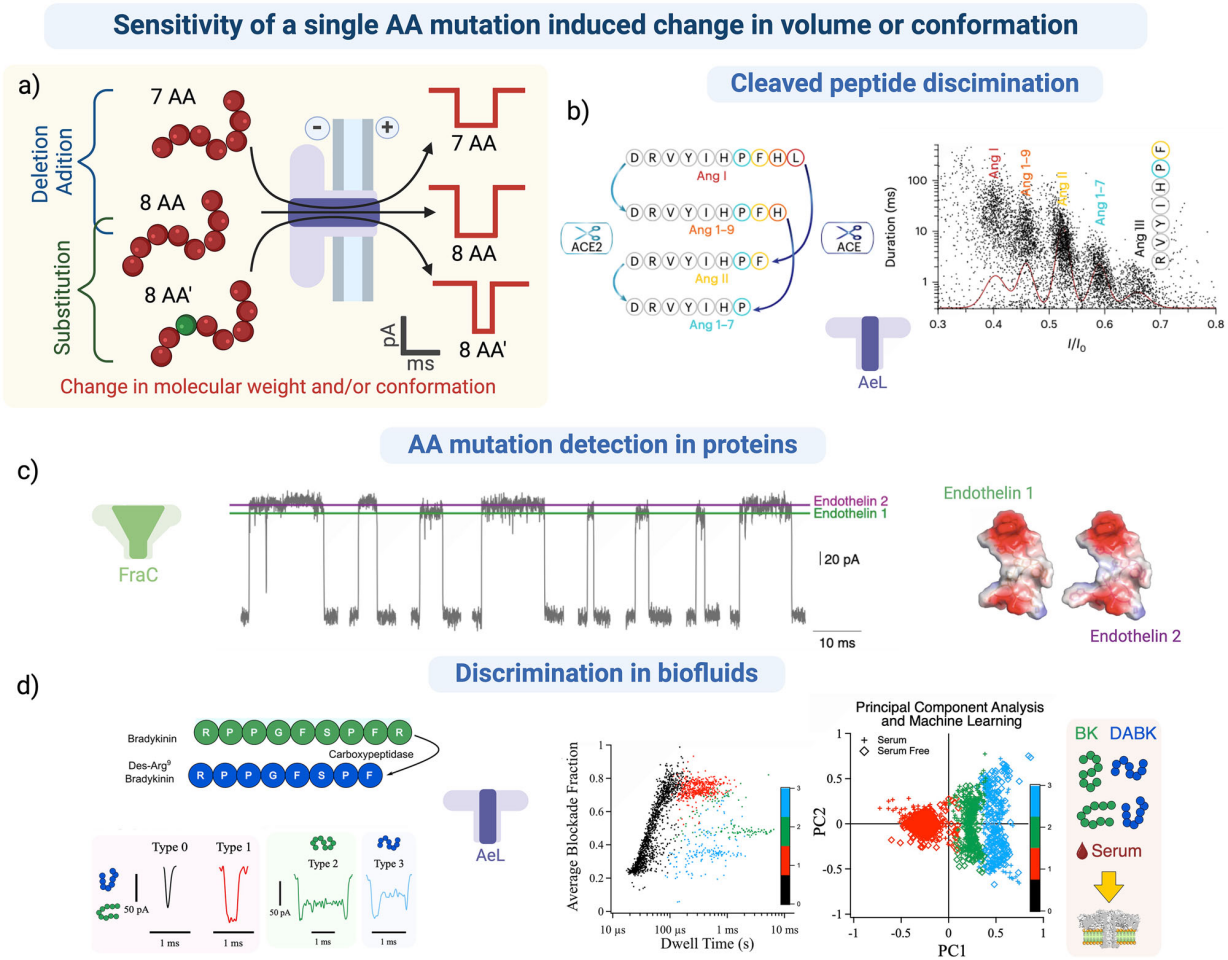
Sensitivity for single amino acid variation-induced changes in volume or conformation. **a** Schematic representation of the characterization of peptides with different types of amino acid mutations such as deletion, addition, or substitution by a nanopore. **b** Identification and discrimination of sequential C-terminal deletion of angiotensin by the enzyme ACE and/or ACE2 (angiotensin-converting enzyme) with an aerolysin nanopore, as defined by the distribution of dwell time (ms) against the mean blockade level (*I*/*I*_0_) in 1 M KCl, 10 mM Tris, 1 mM EDTA, pH 8. Figure adapted from Nature Chem., Jiang et al.^123^. **c** Current traces showing the discrimination between Endothelin 1 and 2 in a mixture with a FraC nanopore in 1 M KCl, 0,1 M citric acid, 180 mM Tris, pH 4,5. Figure adapted from Nature Com., Huang et al.^124^. **d** Discrimination of Bradykinin (BK) and Des-Arginine bradykinin (Des-Arg BK) and identification of different conformations in serum (2%) identified with Principal Component Analysis and machine learning with an aerolysin nanopore in 4 M KCl, 25 mM Tris, pH 7,5. Reprinted (adapted) with permission from Greive, et al. Identification of Conformational Variants for Bradykinin Biomarker Peptides from a Biofluid Using a Nanopore and Machine Learning. *ACS Nano* **18**, 539–550 (2024). Copyright 2024, American Chemical Society^119^. Created in BioRender. Ratinho, L. (2025) https://BioRender.com/t96d726.

The primary sequence of a peptide or protein is crucial for its folding or conformation and activity. Even single mutations, like substitution, addition, or deletion of amino acids, can change the overall folding or the local surface conformation at interaction interfaces that alters the binding to partners or substrates, leading to pathological effects^171^ (Fig. 4a). These changes accumulate with increasing frequency during the aging process, as the cell machinery is less able to detect and correct them^172^.

The utility of nanopore sensing for discriminating between peptides that differ by a single amino acid is well proven^91,92,101,124–126,173–175^. The Long group extended this technology to discriminate between the different peptide substrates and cleaved products of the ACE2 enzyme^123^: Ang I (10 AA), Ang 1-9 (9 AA), Ang II (8 AA), Ang 1-7 (7 AA), and Ang III (7 AA). Each of these peptides were identified simultaneously from a mixed solution depending on the blockade level produced by the relative occupied volume of the peptide confined inside the pore (Fig. 4b) where the variation in volume is related to the peptide length. Angiotensin and its variants are now emerging as relevant biomarkers for cardiac disease, as well as SARS-CoV-2 infections where the ACE2 enzyme appears to be inhibited by the spike proteins^176^. Correct identification of the relative proportions of these variants in a patient could lead to a more accurate diagnosis and better therapeutic management tailored to the phenotype.

In a separate study, a mutated FraC nanopore was also used to identify angiotensin and its variants in a mixed solution depending on the peptide length and the relative volume it occupied in the pore^92^. The mutated FraC permitted the formation of pores of different oligomeric states with varying diameters. The type II FraC nanopore, with a constriction diameter of 1.1 nm, narrower than type I (1.6 nm), increased the dwell time of the confined peptide allowing the identification of a peptide in which a glutamic acid replaced an alanine. These studies on angiotensin highlight the potential of protein nanopores for the accurate, label-free detection and differentiation of angiotensin peptides and variants for clinical diagnosis.

Hemoglobin (Hb) is the preferred biomarker for blood disorders like anemia^177^. The PlyAB nanopore was shown to accurately distinguish between electrical events for adult hemoglobin (HbA), sickle cell anemia hemoglobin (HbS), and fetal hemoglobin (HbF) with over 97% accuracy^32^. The relatively large diameter of PlyAB (7.2 nm) enables protein detection and discrimination, based on their conformation, without the need for unfolding. While the HbA and HbS proteins had similar blockade levels, since they only differ by a single AA substitution, the specific events measured during confinement of each protein fluctuated between two different blockade levels, with the ratio of these levels permitting the discrimination between the two forms of the proteins. In a mixture of HbA, HbS and HbF, the drastically different blockade shapes permitted the discrimination of the 3 proteins. Finally, they demonstrate the counting and identification of Hb directly in blood (0,04% disrupted sheep blood), confirming the potential of this technology for diagnosing pathologies involving Hb and its variants.

The same research group used a WT FraC nanopore to identify several proteins and peptide biomarkers in a mixture^124^. Blockade amplitudes were measured to characterize different biomarker proteins with molecular weights ranging from 25 to 1.2 kDa. Some of these biomarkers varied by only a single amino acid addition or deletion (for example, endothelin I and II, as shown in Fig. 4c) and were discriminated by the local change in conformation, or change in interaction with the pore, caused by the AA mutation, that altered the blockade level and dwell time.

Another group successfully used a MspA nanopore functionalized with a copper ion to detect peptides with a single amino acid variation related to Alzheimer’s disease^93^. This was achieved by sequential hydrolysis of the peptides, where each amino acid underwent reversible coordination with the copper ion bound in the engineered nanopore permitting increased sensitivity and resolution. This method determined the identification of the 20 AA and several PTMs. The discrimination of mutant peptides associated with Alzheimer’s disease is a major research focus, as no effective, rapid, and affordable clinical tool for diagnosis is currently available^178^.

In a recent study, researchers identified variants of a potential biomarker for allergy, cancer, and sepsis in a serum background using an AeL nanopore^119^. They characterized two peptides that differed in length by a single amino acid, bradykinin, and des-9-arg bradykinin. Surprisingly, in addition to the bumping events, each peptide produced two distinct current blockades indicating two possible conformations in solution. Indeed, the confinement of the peptides inside the pore gave sufficient sensitivity and reproducibility in the blockade parameters for the discrimination of these two conformations, even in a background of serum. Machine learning with principal component analysis permitted the identification of 4 types of events depending on their shape. Type 0 events (black; Fig. 4d) correspond to the “bumping” events, which are non-discriminative, and therefore not included in the PCA. Events classified as Type I (red; Fig. 4d) correlate to the expected conformational variant found in both peptides, while those of Types II (green; Fig. 4d) and III (blue; Fig. 4d) are attributed to the alternate conformation. The parameters which are clearly discriminating between the two peptides. Similar blockades were also observed in serum (2% human serum spiked with 50 nM peptides), allowing for peptide identification in a complex biofluid. Indeed, the specific parameters describing Type II and III blockades, arising from the alternative conformational form for each peptide, were essential for identifying the different peptides in mixtures using semi-supervised classification (Fig. 4d). This successfully attributed the type of current blockades to the target biomarker present in serum.

The resolution provided by nanopore technology and the richness of the electrical signals resulting from confinement can accurately distinguish between proteins and peptides, even those with minimal differences in their primary sequence. The unique blockade parameters allow specific discrimination, also in biofluids, highlighting the potential of this technology for rapid and precise diagnosis of diseases that involve protein and peptide variants.

### Post-translational modification detection using nanopore

**Fig. 5:**
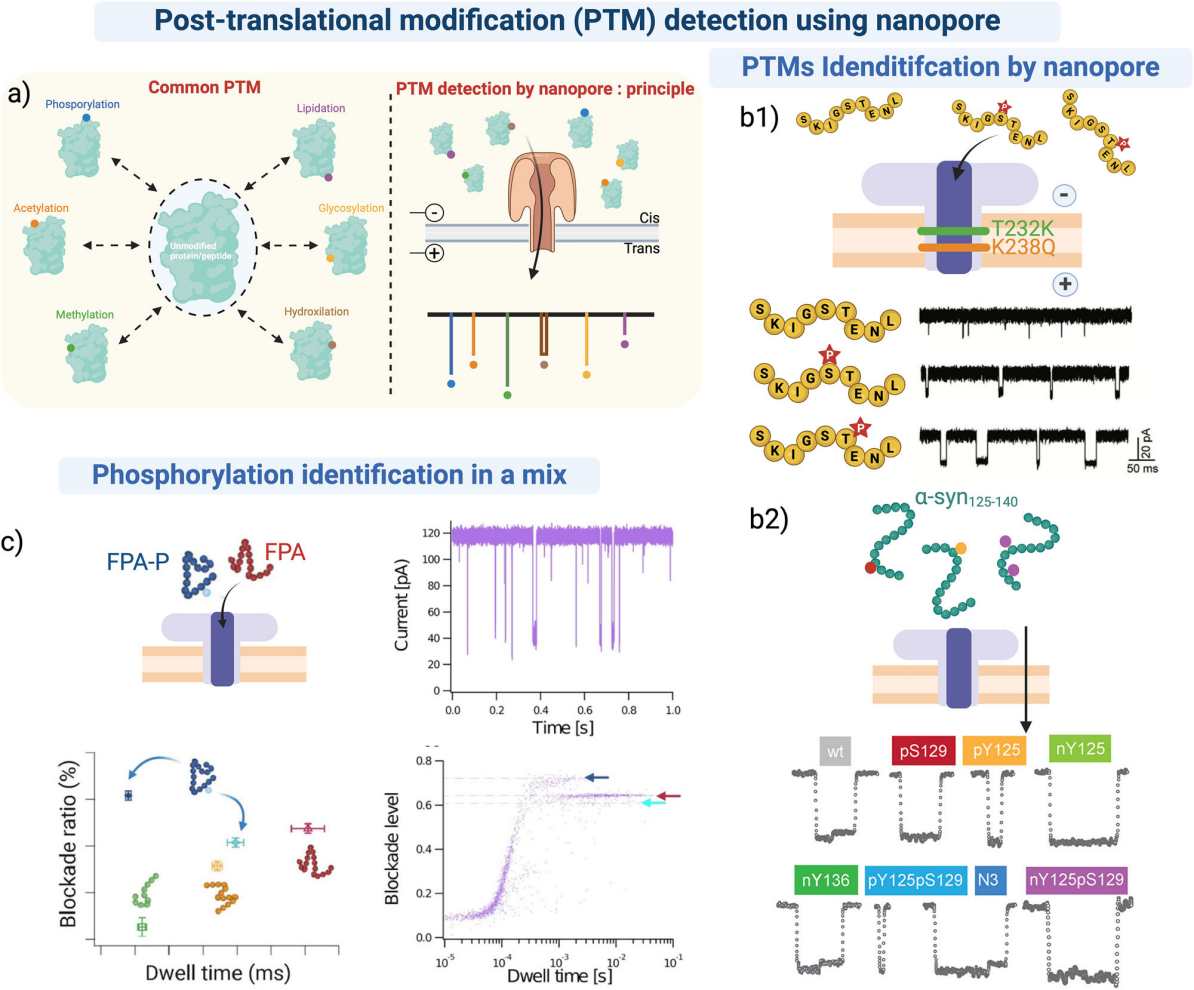
Detection of post-translational modifications (PTMs) by single biological nanopore. **a** Left, sketch representing the most common PTMs. Right, the overall principle of PTM identification using nanopore technology. **b1** Characterization and discrimination of Tau peptides according to different phosphorylation patterns in an engineered aerolysin nanopore (T232K/K238Q) in 1 M KCl, 10 mM Tris, 1 mM EDTA, pH8. Figure adapted from Small Methods, Li et al.^27^ © 2020 WILEY_VCH Verlag GmbH & Co. KGaA, Weinheim^27^ **b2** Current traces showing the identification of different post-translational modifications of α-synuclein peptides with an aerolysin nanopore in 1 M KCl, 10 mM Tris, 1 mM EDTA, pH 7,4. Figure adapted from ACS nano, Cao et al.^127^. **c** Discrimination between unphosphorylated and phosphorylated biological peptides (FPA and FPA-P) with an Aerolysin nanopore. Different blockade levels are observed for each peptide, enabling their identification in the mixture. In addition, due to the high sensitivity of the aerolysin nanopore, two conformations were identified for the phosphorylated peptide. Figure adapted from ACS Central Science, Stierlen et al.^25^. Created in BioRender. Ratinho, L. (2025) https://BioRender.com/j25c673.

Post-translational modifications (PTMs) involve adding functional chemical groups to a peptide or protein^179^. Over 400 PTMs^180^ have been described, with the most common being phosphorylation, acetylation, methylation, glycosylation, and hydroxylation^181^ (Fig. 5a). Of these, phosphorylation is the best studied and linked to physiological processes^182^.

The Bayley group detected and differentiated between the phosphorylation state of thioredoxin on two adjacent sites using an α-hemolysin nanopore^130^. They accomplished this by coupling the C-terminal AA of the protein to an oligonucleotide sequence to increase the EP force for capture and unfolding. The applied EP pulled the C-terminus of the AA chain into the pore constriction, driving the spontaneous unfolding of the rest of the protein that continued to translocate through the pore. The phosphorylation state of the amino acid altered the volume and charge of the unfolded chain at these specific positions, defined by analyzing the blockade parameters of amplitude and noise. This work could lead to using thioredoxin and its PTM-containing variants as biomarkers for cancer and certain neurodegenerative diseases^183,184^. Later, the same research group detected three different PTMs (phosphorylation, glycosylation, glutathionylation) within a large polypeptide using a mutated α-hemolysin nanopore^128^, which increased the EOF for better capture and unfolding of the polypeptide, without conjugation to an oligonucleotide.

An AeL nanopore variant with a double mutation (T232K/K238Q) was used to detect and discriminate between the phosphorylation state of AA at different sites on a peptide fragment of the Tau protein^27^. The authors designed the AeL variant to reduce the translocation speed of the peptide by creating an electrostatic trap inside the channel. This interaction increased the dwell time for confinement of the peptides inside the pore, improving the sensitivity of the pore, enabling the accurate discrimination between the different phosphorylation states of the different amino acids in the peptide sequence (Fig. 5b1). Sensing the phosphorylation state of Tau protein fragments is a diagnostic biomarker for Alzheimer’s disease^185^, as hyperphosphorylation of the Tau protein leads to increased self-assembly and aggregation, implicated in the disease. The same group used an AeL T232K variant, creating an electrostatic trap with the basic ring created by K238 and K232, which resulted in a lower translocation speed. This allowed them to characterize the acetylation and phosphorylation states of peptide fragments of the Tau protein with high sensitivity^95^. They used this new pore to detect and identify the unmodified, mono-acetylated, mono-phosphorylated, and doubly modified peptides. These studies highlight the potential of nanopore technology for the clinical diagnosis of Alzheimer’s disease based on detecting hyperphosphorylation of the Tau protein. The phosphorylation of α-synuclein, like Tau, is involved in another major neurodegenerative disease: Parkinson’s^186^. AeL nanopores were used to differentiate between various combinations of PTMs (phosphorylation, nitration, oxidation) located at different positions in a protein fragment (Fig 5b2)^127^. Additionally, by using blockade parameters (amplitude and duration) in conjunction with machine learning, the authors distinguished between the different variants of α-synuclein with a high level of confidence.

In a recent study, researchers used a MspA nanopore to identify phosphorylation on a threonine within the sequence of the BCAR3 peptide, which is considered a potential cancer biomarker^129^. BCAR3 was linked to an oligonucleotide sequence to drive it into the pore by EP, and the speed of translocation was regulated by a DNA helicase motor (hel308 helicase). This allowed precise measurement of the blockade depth for each AA. The different phosphorylation sites were distinguished by the different blockade amplitudes arising from the increase in relative volume of the pore occupied by phosphorylated compared with unphosphorylated AAs.

One of the most common PTMs on tyrosine is sulfation. This PTM can be linked to the activation of the C-C chemokine receptor type II (CCR2) involved in immune and inflammatory pathways. The Long group exploited the intrinsically charged and hydrophilic residues of the AeL channel, permitting a non-covalent interaction with sulfate groups to identify and discriminate different sulfation patterns of decapeptides derived from CCR2. This proved that nanopore technology is a powerful tool to discriminate between different PTMs, such as phosphorylation or sulfation and could be further implemented to discover tyrosine sulfation patterns in other targets^132^.

Improper DNA compaction by histones can alter transcription regulation, leading to numerous pathologies, including cancer^187^. Nanopore (AeL) technology identified peptides derived from histone 4 that contain hydrophobic methylation or acetylation modifications. The addition of these PTMs increased the overall size of the molecule and potentially increased interaction with the pore through hydrophobic interactions. The ability to discriminate between different isoforms of peptide fragments of the DNA-compacting proteins based on the blockade amplitudes and dwell-times could significantly improve the accuracy of cancer diagnosis^131^. Furthermore, recent advancements have shown the discrimination between unmodified and phosphorylated fibrinopeptide-A peptides in a mixture using an AeL nanopore^25^. Interestingly, this work revealed two different specific blockades for the phosphorylated peptide, indicating two potential conformations in solution (likely flexible unfolded and α-helical; Fig. 5c), compared to just one for the unphosphorylated peptide (likely unfolded).

These studies, which focused on the detection and identification of PTMs present in various biological peptides, demonstrate the sensing power of the unique nanopore generated electrical signals and highlight the potential of nanopore technology as a method for accurate, rapid diagnosis of diseases involving PTMs.

### Enantiomer scale detection by nanopore for diagnosis

Since the discrimination of enantiomers is particularly challenging, especially in a heterogeneous environment, we provide a thorough review of the published work. Studies have shown that enantiomerization may be linked to various pathologies, especially diseases associated with old age. As individuals age, the likelihood of long-lived proteins, such as crystallin in the lens of the eye, undergoing spontaneous enantiomerization of an AA significantly increases^188,189^. Single AAs that act as neurotransmitters can also be subjected to the same process through enzyme activity. The introduction of alternate enantiomers of specific AAs can alter local conformation of peptides or proteins, modulating the affinity of the neurotransmitter or protein for its receptor and other PPI partners, or enhancing self-assembly and aggreagation^188,189^. Detecting AA enantiomers is very challenging since they have identical charge and mass, and may cause only a slight conformational change in proteins or peptides.

**Fig. 6:**
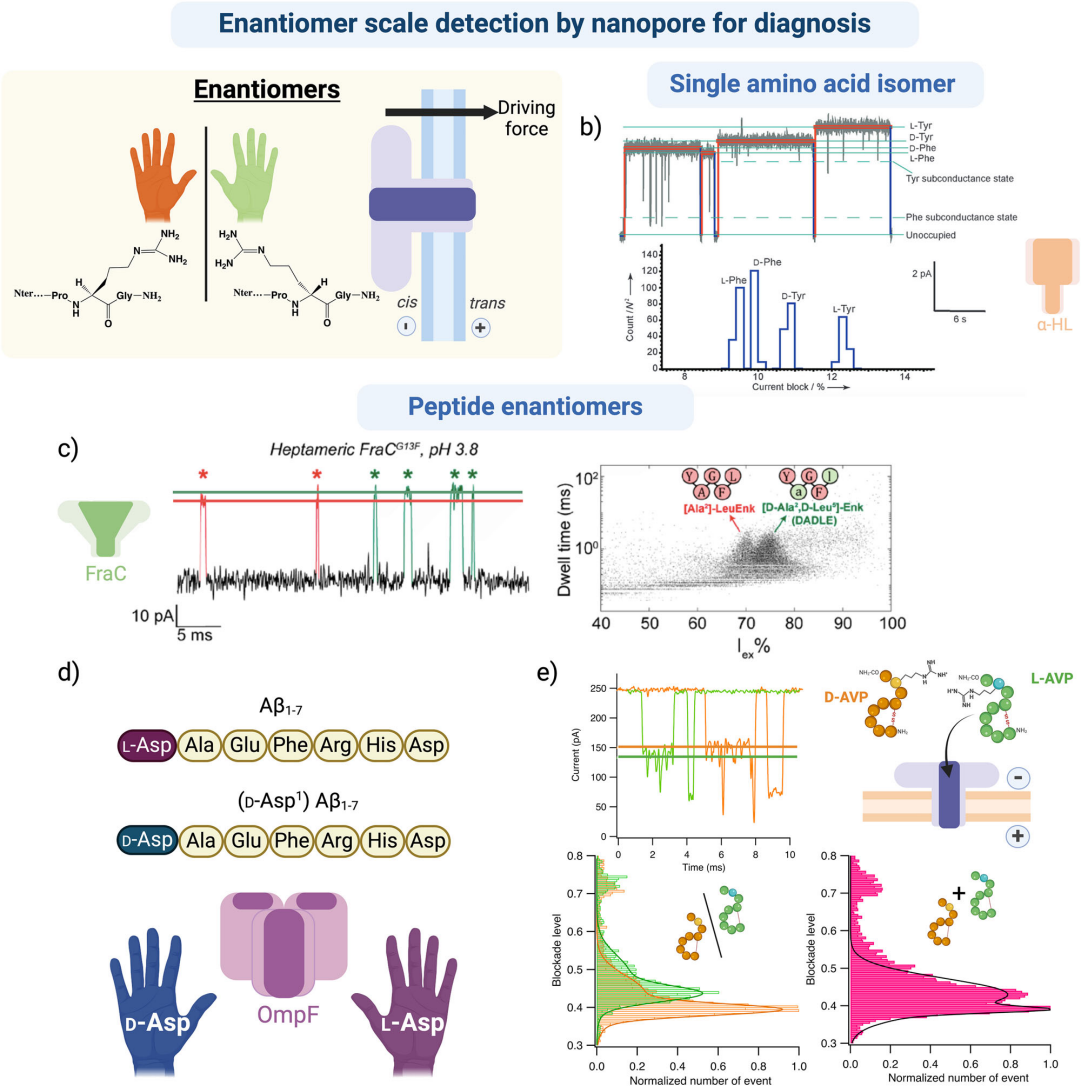
Amino acid scale detection of enantiomer by nanopore for diagnosis. **a** Schematic representation of different forms amino acids as potentially studied by nanopore: structural isomers, enantiomers, rotamers, conformers, and *cis/trans* isomers. **b** Current traces and Current blockade histograms showing the discrimination of dextrogyre and levogyre tyrosine (Tyr) and Phenylalanine (Phe) amino acids with an engineered α-hemolysin nanopore in 1 M KCl, 10 mM MOPS, pH 7.5. Figure adapted from Angew. Chem. Int. Ed., Boersma et al.^190^ © 2012 WILEY_VCH Verlag GmbH & Co. KGaA, Weinheim^190^. **c** Current trace showing the discrimination of two enkephalin peptides that differ by two amino acid enantiomers with a FraC nanopore in 1 M KCl, pH 3.8. Figure adapted from JACS, Versloot et al.^191^. **d** Discrimination of Aβ_1-7_ peptides that differ by one amino acid enantiomer with an OmpF nanopore^193^. **e** Current traces of L-Arginine and D-Arginine Vasopressin (L- and D-AVP) and the resulting histogram of blockade levels showing the characterization and discrimination of the two peptides in an equimolar mixture with an aerolysin nanopore in 4 M KCl, 25 mM Tris, pH 7.5. The histogram of the blockade levels shows multiple populations representative of a change in conformation or orientation of the peptides—figure adapted from ACS Cent. Sci.; Ratinho et al.^118^. Created in BioRender. Ratinho, L. (2025) https://BioRender.com/k79m010.

The conformational sensitivity of nanopore sensing can detect enantiomer differences and may, eventually, link them to pathologies (Fig. 6a). Due to the difficult nature of these assays, only a few papers have, been published to date that demonstrate discrimination between chiral AAs.

The first such work on single AAs showed excellent discrimination between L- and D-Cys, Phe, Asp, and Trp using an engineered copper-bound α-hemolysin nanopore that transiently bound to the amino acids^190^ (Fig. 6b). Guo et al. used a similar technique with an α-hemolysin nanopore and a copper-complexed cyclodextrin. They were able to discriminate between all the single Aromatic Amino Acid (AAA) enantiomers in a mixture, depending on their blockade levels. However, discrimination between AA enantiomers in biological peptides and proteins is an ongoing challenge.

Work from the Maglia group used two engineered nanopores: CytK WT, K128F, K128D, with a cylindrical β-barrel conformation, and FraC WT, G13F, an α-helical pore with a more conical shape. These mutations permitted the addition of aromatic or acidic sensing regions which likely interacted with the analytes, increasing the confinement time of the peptides inside the pore. Despite differently shaped chambers, both pores were able to discriminate between 2 enantiomeric homopolymer peptides in a mixture, with a similar degree of resolution^191^. This was extended to show discrimination between enantiomeric forms of a biological peptide: the delta opioid peptide [Ala2] Leu-enkephalin and its variant, [D-Ala2, D-Leu5] enkephalin^192^. Both CytK and FraC nanopores could discriminate between the two biological peptides based on their blockade levels (Fig. 6c).

The Long group used an OmpF nanopore to discriminate between two Aβ peptides that differed by a single AA enantiomer. OmpF has an electrostatically asymmetrical constriction of 0.7 nm, close to the diameter of a single AA. This was predicted to enhance AA scale sensitivity and help discriminate between the enantiomers based on their location in the peptide chain and the orientation in relation to the peptide backbone. Indeed, the researchers were able to discriminate between Aβ peptides, differing in L- or D-Asp^193^ (Fig. 6d).

The same group engineered an heteromeric AeL nanopore where binding of maleimide derivative to the 3:4 K238C AeL monomer created a stereo-regio-specific heteromeric nanopore containing an asymmetric hydrophobic clamp structure defined by the orientation of the phenyl groups from the maleimide derivatives. These changes increased the blockade level and dwell time of the events, permitting accurate discrimination of synthetic peptides bearing a single enantiomeric amino acid^194^.

Finally, the Cressiot and Pelta groups used an aerolysin nanopore to discriminate between two peptide biomarkers that differed by a single AA enantiomer^118^. L-arginine vasopressin (L-AVP) is a potential biomarker of diabetes insipidus^195^, and D-arginine vasopressin (D-AVP) is a drug analog used as a diuretic agonist^196^. Discrimination between those two peptides is complicated by the presence of a disulfide bond, which constrains the peptide into one of two native conformations in solution: the open and saddle forms. The latter form is further constrained by the presence of two hydrogen bonds within the peptide loop. This work was not only able to distinguish between these two conformations, but also to correctly determine the 70/30 ratio between them, consistent with previous NMR data^197^. Additionally, this work clearly distinguished each L- and D-AVP peptide from a mixture in both native and reduced conditions (Fig. 6e).

This work proves that nanopore sensing is sufficiently powerful to distinguish between peptides that differ by a single enantiomeric AA, one of the most challenging discrimination problems for which there are no other quantitative methods. Modulation of nanopore systems to increase the confinement time inside the pore allows discrimination of single AAs and peptides depending on their enantiomer state. Indeed, this technology could be an asset in establishing a new POC diagnosis assay capable of quantifying peptides and/or their enantiomer state at low concentrations.

## Conclusion

Protein and peptide biomarkers are an important area of development in clinical diagnosis and health management, especially with the increasing prevalence of diseases associated with an aging population^1^. Clinical assays capable of detecting very low concentration biomarkers are particularly important for early diagnosis when biomarker levels are slightly altered. As we learn more about how small changes in protein/peptide shapes affect interactions that maintain homeostasis, and how these changes are disrupted in disease, it’s likely that measuring these shape changes will become an important part of clinical tests for new biomarkers. Many of these structural changes result from modifications of one or more amino acids in the protein or peptide sequence, the presence of PTMs, or the inclusion of different enantiomers. Currently, these changes are primarily studied using mass spectrometry, CryoEM, X-ray diffraction, or fluorescence methods, providing invaluable data on the conformational variety for potential biomarkers. However, these methods are unsuitable for use in clinical settings that require quantitative, rapid, sensitive and cost-effective tests for diagnosis^198^. While immunoassays are routinely used in many clinical and POC assays, these are not always able to detect very low levels of such subtle conformational differences. As highlighted in this review, nanopore technology has significant potential for application in clinical diagnosis; not just for understanding the fundamental biophysical properties of these biomarker analytes, but also in detecting and quantifying changes in peptides or proteins that are relevant to health.

Nanopore sensors are uniquely suitable for quantitative analysis of conformational variation in proteins and peptides. This arises from the combination of single-molecule sensing with the feature rich signals that can discriminate between analytes with different PTMs^128^, single AA substitutions^123^, or enantiomeric AAs^118^. Careful concentration calibration, tuning of the driving forces and event classification methods can quantify different populations from 10^2^ to 10^3^ molecules^60^,^188^, across a broad range of analyte concentrations. Despite this, numerous challenges remain to be solved before this technology can be widely used for protein/peptide analysis in biomarker research, let alone for diagnosis in clinical settings.

One of the main challenges for the quantitative analysis of biomarker variants is that many of these potential biomarkers are present in biofluids at very low concentrations^15,16^. Despite the single-molecule resolution, the frequency with which an analyte interacts with the pore, and therefore the assay time, is dependent both on the analyte concentration^75,99^ and the driving force^89^. The requirement for electrolyte solutions to mediate these driving forces means that patient samples will always be subject to dilution, further reducing analyte concentration. However, as described above, the driving forces for analyte-pore interaction can be used to increase capture efficiency in numerous ways. Indirect detection can increase the EP driving force on the target analyte by using heavily charged tags or binding partners. In addition to selectivity introduced by specific binding of the analyte to the probe, indirect detection can also be multiplexed with DNA barcodes^199^ and DNA probes^200,201^. For instance, using DNA barcodes in a biological nanopore it was possible to simultaneously identify five different cancer biomarkers in the picomolar range^202^. However, indirect detection requires additional preprocessing steps and is dependent by the inherent limitations of the binding partner to differentiate between different forms of the same biomarker. While nanopore confinement of the bound analyte can overcome this problem with discrete signals that discriminate between different biomarker forms, a label-free direct detection method is superior. The capture efficiency in direct detection can be improved by increasing the EOF driving force. This can be achieved by altering the ion composition of the electrolyte solution or creating a concentration gradient of ions across the nanopore in the direction of the EOF^87,89,100,203^. However, the most common method to increase the EOF is by engineering the protein nanopore such that the pore selectivity for a particular counterion is augmented^27,106,107,204^. This could be designed so that the charge of the pore-selected ion is the same polarity as the analyte charge, making the EP and EOF synergistic. Finally, recent work has explored using molecular crowding agents to increase the frequency of analyte interaction with the pore^205,206^. Indeed, this may represent the most promising solution to the detection of low-concentration biomarkers.

Sensing resolution can also be improved by increasing the interaction time of the analyte with the pore. This can be achieved by increasing confinement and modulating the EOF, through protein engineering, often coupled with bioorthogonal chemistry^190,207^. The use of engineered asymmetric pores has enhanced differentiation between chiral amino acids^193,194^. Improving signal-to-noise ratio in analyte sensing is vital for defining each unique event with as many parameters as possible. In addition to the widely used blockade parameters of *I*_b_, *T*_t_, and *σ*, additional shape features such as skewness or kurtosis, sub-level analysis (number, amplitude, and duration), and event entry and exit slopes (right and left) provide detailed information for event classification^25,208–212^. The confidence in identifying a specific biomarker from a mixture increases with each additional unique current signal which often arise from conformational variations and appear in defined relative proportions. This was demonstrated in a recent study, where a combination of the AeL nanopore and supervised classification, identified the presence of different conformations for two similar biological peptides in a serum background^119^. While machine learning has proven useful for identifying specific biomarkers from the background of blockade signals generated from mixtures of biomarkers in biofluids, the true power of artificial intelligence (AI) is unleashed by the large and varied datasets used to train the classification models. This was demonstrated by the success of AI generative and predictive algorithms such as Large Language Models and Alphafold, trained on large text libraries and the Protein Data Bank, respectively^213,214^. Consequently, the main roadblock to using AI algorithms in nanopore sensing and identification of different biomarkers is the lack of training data. An additional problem in developing a database of analyte blockade parameters is that blockade features are unique to each analyte-pore pair. Indeed, parallel sensing of specific analytes with multiple pore variants would increase the number of specific blockades associated with different forms of the biomarker. However, as the library of identifying blockade parameters increases for each analyte-pore combination, the accuracy of AI-driven classification will only continue to improve, such that, with time, multiplexed quantitative detection of low concentrations of different biomarkers, and their different forms will be possible.

While nanopore assays are unlikely to replace currently available POC tests, there are key areas where new technology would significantly improve health management. Current clinical tests that take several days create a delay in treatment that might lead to poor outcomes in high-risk patients. For example, neuraminidase drug resistance screening for influenza uses high numbers of virions, requiring a culture step between sample collection and the assay. Nanopore sensing could potentially reduce the assay time to hours, allowing earlier treatment. Time-sensitive treatment decisions are vital in situations where health can deteriorate quickly, such as for stroke, heart-attack, and bacterial meningitis. In such cases, early detection, when the biomarker is marginally altered, could significantly improve treatment. Use of nanopore sensing would potentially facilitate quantitative detection of specific biomarkers, such as brain natriuretic peptides for cardiovascular function, at the very low levels that exist early in the medical crisis, allowing prompt treatment^152^.

Nanopore devices for use in routine medical research will require robust platforms supporting arrays of nanopore sensors that are stable in high concentrations of biofluids, to minimize sample processing. Most current nanopore protein/peptide biomarker detection technology is limited due to fragility of the lipid membranes. This also limits the use of crowding agents or electrolyte gradients to increase the capture frequency. Production difficulties hinder scale-up for use in clinical applications^215^. Moreover, the complex composition of biofluids presents several challenges for lipid membranes, which are sensitive to osmotic pressure. Additionally, biofluids often contain lipid-permeable or disruptive components^216^. One possible solution is to use synthetic polymers, which are more robust than lipid membranes^217^. Another approach involves creating hybrid nanopores^218^, which combines the stability of a solid state nanopore with the near-atomic scale tunability of protein nanopore adapters. Indeed, protein nanopores engineered to increase sensing sensitivity can be used as the inserted hybrid adapter^105^. Finally, after overcoming all these limitations, studies on cohorts of patients to define baseline and measurement levels of each biomarker form will be required as a final validation step before the technology can be implemented in a clinical setting.

The final challenge to using nanopore sensing as a POC device is the need to combine nanopore technology with nanofluidic systems and electronic detection that is robust, sensitive, and miniaturized, like the Minion DNA sequencer from Oxford Nanopore Technology. POC devices will require minimal sample processing and user-friendly software on portable electronic platforms. Such software, loaded with pretrained AI algorithms allowing both detection and interpretation of blockade signals into clear diagnostic measurements will provide a robust, portable device for POC health management.
